# Psychosocial Impact of Virtual Cancer Care through Technology: A Systematic Review and Meta-Analysis of Randomized Controlled Trials

**DOI:** 10.3390/cancers15072090

**Published:** 2023-03-31

**Authors:** Caterina Caminiti, Maria Antonietta Annunziata, Paola Di Giulio, Luciano Isa, Paola Mosconi, Maria Giulia Nanni, Michela Piredda, Claudio Verusio, Francesca Diodati, Giuseppe Maglietta, Rodolfo Passalacqua

**Affiliations:** 1 Clinical and Epidemiological Research Unit, University Hospital of Parma, 43126 Parma, Italy; 2 Oncological Psychology Unit, Centro di Riferimento Oncologico di Aviano (CRO) IRCCS, 33081 Aviano, Italy; 3 Department of Public Health and Pediatrics, University of Turin, 10124 Torino, Italy; 4 Division of Oncology, Hospital of Melegnano, 20064 Gorgonzola, Italy; 5 Laboratory for Medical Research and Consumer Involvement, Department of Public Health, Istituto Di Ricerche Farmacologiche Mario Negri IRCCS, 20156 Milan, Italy; 6 Department of Neuroscience and Rehabilitation, Institute of Psychiatry, University of Ferrara, 44121 Ferrara, Italy; 7 Research Unit of Nursing Sciences, Campus Bio-Medico University of Rome, 00128 Rome, Italy; 8 Department of Medical Oncology, Presidio Ospedaliero di Saronno, ASST Valle Olona, 21047 Saronno, Italy; 9 Medical Oncology Division, Department of Oncology, ASST of Cremona, 26100 Cremona, Italy

**Keywords:** cancer, telemedicine, virtual care, psychosocial health, quality of life, anxiety, psychological distress

## Abstract

**Simple Summary:**

Modern technology now enables the remote delivery of cancer care, often called telemedicine. Despite the many potential advantages, it is still not known whether a lack of in-person contact with clinicians may affect the psychosocial health of cancer patients. This systematic review and meta-analysis aimed to determine whether totally or partially replacing conventional face-to-face hospital care with telemedicine deteriorates quality of life, anxiety, psychological distress, or depression of adult people with cancer. The results of the eight included randomized trials seem to indicate that telemedicine does not worsen psychosocial health. On the contrary, we observed improvements in favor of telemedicine in all considered outcomes. Possible explanations are that technology improves access to information and facilitates contact with clinicians, and that being followed at home reduces uneasiness. Future research should identify which patients may benefit from telemedicine, and those for whom traditional in-person visits remain the best option.

**Abstract:**

This meta-analysis of RCTs aimed to determine whether replacing face-to-face hospital care with telemedicine deteriorates psychosocial outcomes of adult cancer patients, in terms of quality of life (QoL), anxiety, distress, and depression. RCTs on interventions aimed at improving patient psychosocial outcomes were excluded. MEDLINE, EmBASE, and PsycInfo were searched on 13 May 2022 without language or date restrictions. In total, 1400 records were identified and 8 RCTs included (4434 subjects). Study methodological quality was moderate. Statistically significant improvements were observed in favor of the intervention for QoL (SMD = 0.22, 95% CI 0.01 to 0.43, *p* = 0.04), anxiety (SMD = −0.17, 95% CI −0.30 to −0.04, *p* < 0.01), and global distress (SMD = −0.38, 95% CI −0.51 to −0.25, *p* < 0.01). A meta-analysis on depression could not be performed. In subgroup analyses, the intervention appeared to be more beneficial for patients receiving active treatment vs. follow-up, for “other cancer types” vs. breast cancer, and for “other modes of administration” vs. telephone. Given the many potential advantages of being assisted at home, telemedicine appears to be a viable option in oncology. However, more research is necessary to determine the types of patients who may benefit the most from these alternative care modalities.

## 1. Introduction

In-person care has traditionally been considered the “gold standard” of interaction between patients and physicians [1]. However, with the widespread availability of new communication technologies, such as computers and smartphones, alternative ways of providing assistance at a distance are emerging. Various terms such as telemedicine, telehealth, virtual care, and others are now frequently and interchangeably used to refer to remote access to health care, health information, or health education through technology [2]. The use of telemedicine has recently grown exponentially due to the prolonged COVID-19 pandemic, as it enabled a continuity of care while maintaining social distancing, avoiding unnecessary hospital visits, or reducing time spent in health care facilities [3,4]. This was particularly crucial for cancer patients, who are at an increased risk of contracting COVID-19, and if infected with COVID-19, have worse outcomes [5,6,7]. Despite the role of the pandemic in promoting the adoption of virtual care in routine practice, its ongoing use suggests that it may remain an integral part of cancer care delivery [8].

Telemedicine can offer various advantages over traditional in-person care, including overcoming barriers to healthcare access such as distance to hospitals, travelling cost, and time restraints, with less disruption to family life [9,10]. Furthermore, from an organizational point of view, there have been several calls within oncology that the current model of traditional in-person care delivery by the specialist team is unsustainable as the population of cancer patients and survivors grows [8]. However, the use of telemedicine could generate inequality in the provision of healthcare to people with different digital health literacy or with limited access to technology [11,12]. Virtual visits must continue the best aspects of in-person care to be an acceptable substitute for it [13]. Therefore, the different pros and cons should be taken into account before offering this option to patients. One essential element to consider is the possible impact that the provision of care without in-person interaction may have on patient psychosocial health. This is particularly relevant for people with cancer, whose psychosocial symptoms, though highly prevalent, are often unrecognized and untreated [14,15,16], with negative consequences on the patient’s coping, adherence to therapy, social and family relationships, quality of life (QoL), and survival [14,17]. In this view, it is essential to examine whether the emerging technology-based modes of healthcare provision may influence the psychosocial health of this vulnerable population.

Numerous literature reviews on the impact of virtual care on patient psychosocial health have been published [1,18,19,20,21,22,23,24,25,26,27,28]; however, they generally apply broad eligibility criteria to include a wide range of interventions provided remotely, not restricted to routine care, but extended to telehealth-based psychosocial treatments (e.g., psychological support, information, advice or self-management strategies, lifestyle modification programs, etc.).

We thus conducted this systematic review and meta-analysis to determine whether totally or partially replacing face-to-face hospital care with telemedicine deteriorates the psychosocial health of adult people with cancer. Predesigned subgroup analyses were also performed according to phase of care, cancer type, and mode of intervention delivery.

The review questions were the following:

1. Does telemedicine, compared to in-person care, negatively affect patient psychosocial health in adult cancer patients?
2. Do the psychosocial effects of the interventions change according to the phase of care (active treatment vs. follow-up)?
3. Are there differences in the size of the effects based on the type of cancer?
4. What modes of intervention delivery work best?

## 2. Methods

### 2.1. Overview

The idea for this research is based on the work of the Panel for the Guidelines on the Psychosocial Care for Adult Cancer Patients, published by the Italian Association of Medical Oncology (AIOM) annually since 2012 [29]. In light of the unprecedented rise in virtual care in oncology following COVID-19, the panel decided to investigate the psychosocial impact of new forms of technology-based distance care, in order to include corresponding recommendations in the next guideline update. In particular, it was the panel’s concern that a lack of in-person contact may negatively impact the psychosocial health of a vulnerable population, already at higher risk for psychosocial problems. This paper describes the methodology followed for the systematic review conducted for this purpose and reports the quantitative analyses of results of the identified randomized trials.

Before conducting this work, in April 2022, the International Prospective Register of Systematic Reviews (PROSPERO) [30] was searched to make sure that no review investigating the same study questions was underway, in order to avoid replication, but none was found. This systematic review was designed and conducted following the Preferred Reporting Items for Systematic reviews and Meta-Analyses (PRISMA) guidelines [31]. It was registered on PROSPERO under the number CRD42022321716.

### 2.2. Eligibility Criteria

Only randomized controlled trials (RCTs) were considered, because study designs without randomization imply a higher risk of bias and the estimation of causal effect is more difficult. Non-randomized trials and observational studies were therefore excluded, as well as editorials, commentaries, methodological articles, letters to editors, and case reports. A literature search according to the population, intervention, comparison, outcome, and timing (PICOT) model was performed, as recommended by PRISMA [31]. The criteria for study selection were:

Population: Adult (age ≥ 18 years) patients who had received a diagnosis of any type of cancer, in any phase of the care trajectory (covering all aspects of follow-up and survivorship). Studies on children, adolescents, or young adults were excluded.

Intervention: Virtual care replacing, totally or in part, in-person care, which is generally offered face-to-face in clinical practice during visits with clinicians. Trials where the intervention consisted of a combination of telemedicine and in-person care were also considered. Interventions could use different modes of delivery, such as telephone calls, email, smartphone apps, or teleconferencing. We thus excluded studies on interventions primarily aimed at improving patient psychosocial outcomes, such as psychotherapy and other allied health care services, as well as educational or lifestyle interventions. We also excluded trials comparing virtual care interventions in both arms.

Comparator: Eligible RCTs had to include a usual care arm, consisting in the provision of healthcare exclusively face-to-face by a clinician (physician or nurse). RCTs comparing two forms of telemedicine (e.g., telephone vs. teleconferencing), without an in-person arm, were excluded, as the aim of this work was to assess any difference between virtual and in-person care in terms of impact on psychosocial health.

Outcomes: Trials had to investigate the effects of telemedicine on patient psychosocial outcomes and include Patient-Reported Outcome Measures (PROMs) detected using validated instruments. Trials had to include at least one of the following psychosocial outcomes: QoL, anxiety, depression, and global distress, measured as post-intervention variables.

### 2.3. Selection Process

The MEDLINE (PubMed), EmBASE, and PsycInfo databases were searched on 13 May 2022. No date or language restrictions were applied. A “backwards” snowball search was conducted of the references of systematic reviews and of included articles. Full search terms and search strategies for the three databases are given in Supplementary Table S1.

Two reviewers (CC and FD) independently performed the initial title and abstract screening for relevance to the review using the Rayyan platform [32], which allows them to record any discrepancy and facilitates the agreement process. Eligibility criteria for population and intervention could mostly be ascertained at this stage. Next, two reviewers (PDG and MP) independently examined the full texts of publications identified as potentially eligible and performed study selection. The presence of suitable outcomes and availability of data were verified. Any disagreements between individual judgments were resolved by a third independent reviewer (CC).

### 2.4. Data Extraction

Two reviewers (CC and GM) independently extracted data from selected trials using a Microsoft Excel form, and disagreements were resolved through discussion. Extracted data items included: title and first author, country, number of centers, cancer type, number of randomized patients, phase of care (active treatment or follow-up), intervention delivery method, outcomes and corresponding instruments, estimates of the effect, and measures of variability (standard errors or confidence intervals).

### 2.5. Risk of Bias (Quality) Assessment

We assessed the risk of bias of the included RCTs using the Cochrane risk of bias tool for randomized trials (RoB 2) [33] and the criteria specified in Chapter 8 of the Cochrane Handbook for Systematic Reviews of Interventions [34]. The methods followed in this study are described in Appendix A.

### 2.6. Assessment of Reporting Biases

The protocol included the use of funnel plots to assess reporting biases (such as publication bias). However, this was not possible, as we were not able to pool more than 10 trials [35].

### 2.7. Data Synthesis

We could not undertake the meta-analysis for one outcome (depression), which was only considered by one RCT. For the remaining outcomes, we provided the description of the results using a forest plot. For the meta-analysis, we combined continuous data from psychosocial outcomes scales that were sufficiently similar using generic inverse variance and standardized mean difference (SMD), also known as Cohen’s d, to account for differences in the scales [34]. The values of SMD of 0.2, 0.5, and 0.8 are considered small, medium, and large, respectively [34,36]. We used a random-effects model for the meta-analysis to account for possible differences among studies in which conditions of the health care setting and approach may have varied. We computed the meta-analysis by using R-Cran Statistical Software v 4.2.2 with meta and metafor packages.

### 2.8. Subgroup Analysis and Investigation of Heterogeneity

If a sufficient number of studies were available, subgroup analyses were planned to explore the following study characteristics as sources of heterogeneity: cancer type, phase of care (active treatment and follow-up), and modes of intervention delivery. Significance was defined as *p* < 0.05.

The heterogeneity was assessed using the I2 (percentage of between-study variance due to heterogeneity rather than sampling error) statistics test. Thresholds for the interpretation of the I2 according to the Cochrane Handbook [34] are as follows: 0% to 40%: might not be important; 30% to 60%: may represent moderate heterogeneity; 50% to 90%: may represent substantial heterogeneity; and 75% to 100%: considerable heterogeneity.

## 3. Results

### 3.1. Study Selection 

A total of 1400 articles was retrieved from the three databases and uploaded into the Rayyan platform. After the removal of duplicates, 1280 records underwent title and abstract screening. We chose to perform this task manually without applying automation tools to increase accuracy. Nineteen reports were deemed potentially eligible and underwent full text review. Of these, thirteen [37,38,39,40,41,42,43,44,45,46,47,48,49] were excluded, because telemedicine constituted an addition to, and not the partial or total replacement of, usual care, because comparison was made between two forms of telemedicine, or because outcomes of interest for the review were not investigated (Supplementary Table S2). Six papers [50,51,52,53,54,55] were selected. Finally, two additional eligible studies were identified, one from the reference list of a systematic review [56] and one from the reference list of a study [57]; therefore, a total of eight reports were included in the review.

**Figure 1:**
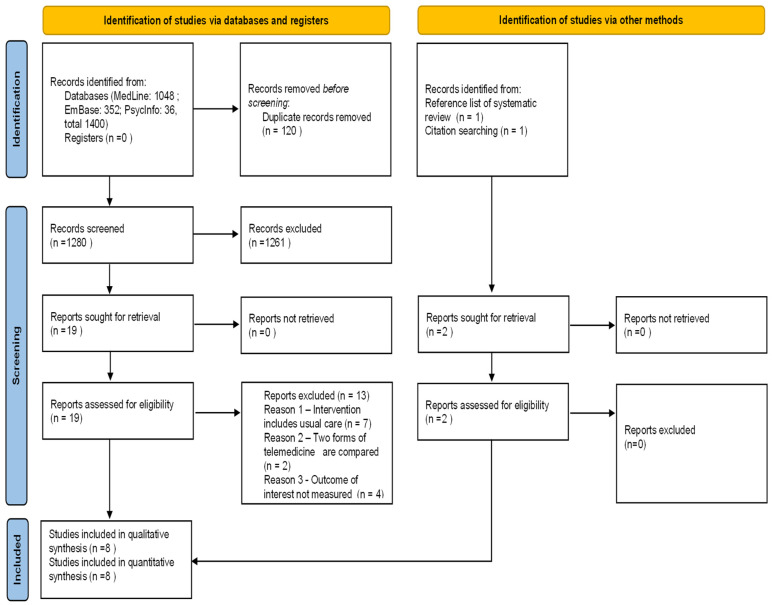
PRISMA 2020 flow diagram. Flow diagram of identified studies, included and excluded [31].

A flow diagram depicting the selection process is provided in Figure 1.

### 3.2. Study Characteristics 

The RCTs evaluated 4434 patients (2090 intervention arm vs. 2344 control arm), with 94% (4185/4434) being women. The time of publication ranged from January 2009 to November 2021. Five of the selected studies were based in Europe, one in the USA, one in Canada, and one in China. The mean age of the study participants was 60 years. Regarding cancer types, there were 2507 women with breast cancer, 474 with endometrial cancer, 170 with ovarian cancer, 50 patients with colorectal cancer, 80 with head and neck cancer, and 829 patients in the Maguire study [54] receiving chemotherapy for non-metastatic breast cancer, colorectal cancer, or non-Hodgkin’s lymphoma. We analyzed the data of 1493 patients (five studies) for the measure of effect on QoL, 2005 (seven studies) for anxiety, 416 (two studies) for global distress, and 580 (one study) for depression. The scales used to measure anxiety were the state-trait anxiety inventory (STAI) [50,51,52,54,56], the Hospital Anxiety and Depression Scale (HADS) [57], and the Generalised Anxiety Disorder 7 Scale (GAD-7) [53]. QoL was measured with the EORTC Core Quality of Life Questionnaire (EORTC QLQ-C30) [52,56,57], a specific module for endometrial cancer (QLQ-EN24) [56], the Functional Assessment of Cancer Therapy—General (FACT-G) [54], the Functional Assessment of Cancer Therapy-Head & Neck Scale (FACT-HN) [55], and the EuroQol EQ-5 Dimensions-3 Level (EQ-5D-3L) [53]. Global distress was assessed with the Global Distress Index (GDI) of the Memorial Symptom Assessment Scale (MSAS) [54,55]. Finally, the scale for measuring depression was the Patient Health Questionnaire 9 Scale (PHQ-9) [53]. Intervention groups that received technological-based interventions were compared with control groups that received care as usual at the cancer center (consultation, clinical examination, etc.). Five out of eight interventions [50,51,52,56,57] addressed patients in follow-up, consisting in telephone consultations led by specialist nurses. The remaining three studies concerned patients receiving active treatment, followed for monitoring and management of toxicities. Among these studies, mode of delivery varied (telephone [53], website [54], and telehealth messaging device [55]). All studies used multiple impact measures, but none examined the effect of telemedicine on all four outcomes of interest for this review. Specifically, global distress was reported in two studies (as a primary outcome in Pfeifer et al. [55] and a secondary outcome in Maguire et al. [54]), anxiety was considered in seven studies (as a primary outcome in the three Beaver studies [50,51,56] and as a secondary outcome in Kimman et al. [52], Krzyzanowska et al. [53], Maguire et al. [54], and Ngu et al. [57]), depression was evaluated only as a secondary outcome in one study [53], while QoL was a primary endpoint in two studies [52,55] and a secondary endpoint in four [53,54,56,57]. A summary of characteristics of the eight studies included in the review is provided in Table 1.

### 3.3. Risk of Bias in Studies 

**Figure 2:**
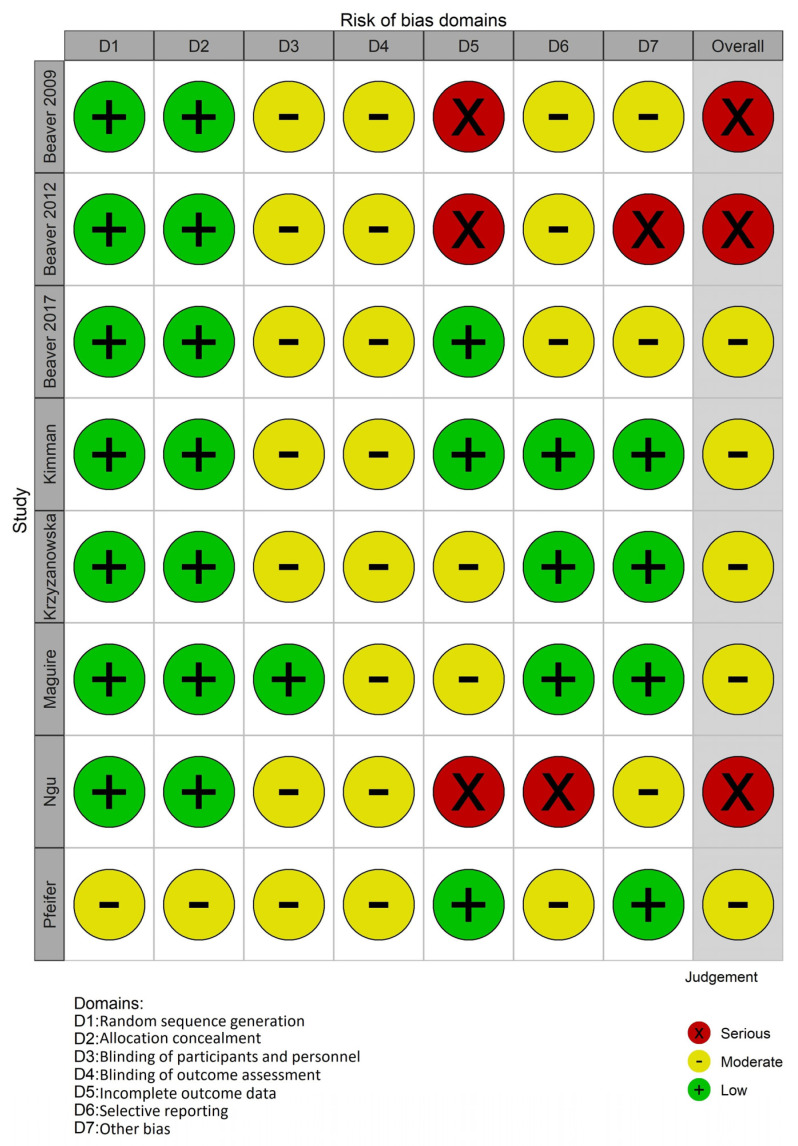
Risk of bias graph: review authors’ judgments about each risk of bias item presented as percentages across all included studies.

**Figure 3:**
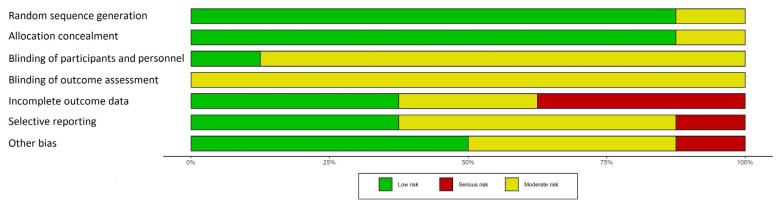
Risk of bias summary: review authors’ judgments about each risk of bias item for each included study.

We provide a “risk of bias” graph (Figure 2) with the review authors’ judgments about each “risk of bias” item presented as percentages across all included RCTs. We also provide a “risk of bias” summary (Figure 3), with the review authors’ judgments about each “risk of bias” item for each included study.

The risk of bias is detailed for each individual study in Supplementary Table S3 and summarized below.

***Randomization process***

Only one of the eight trials [55] was rated as having an “Unclear” risk of selection bias, as the reported information was insufficient to make a judgment about sequence generation. The other seven studies clearly specified sequence generation and were assessed as having a “low” risk of bias, as no baseline imbalances were apparent between groups to suggest a problem with the randomization process.

***Allocation concealment***

Seven studies gave information about whether the allocation sequence was concealed until participants were enrolled and assigned to the intervention [50,51,52,53,54,56,57]. Only the Pfeifer study [55] did not provide sufficient details and therefore was judged to exhibit some problems.

***Blinding of participants and personnel***

Performance bias refers to systematic differences between groups in the care that is provided or received and requested [34]. Only one of the studies [54] provided information on whether the participants were aware of the intervention assigned during the study. However, the nature of the intervention makes it very likely that the participants were aware of the intervention assigned to them during the process. We judged all studies to have an “unclear” risk of bias, as blinding was either not possible or not reported.

***Blinding of outcome assessment***

Detection bias refers to systematic differences between groups in how outcomes are determined [34]. Study participants were the outcome assessors, and since outcomes were self-reported, they were probably aware of the intervention status. While outcome assessment may have been influenced by the knowledge of the intervention received, there is no strong reason to believe that it did. We rated all studies as having an “unclear” risk of detection bias.

***Incomplete outcome data***

Attrition bias refers to systematic differences between groups in withdrawals from a study [34]. We judged three studies [52,55,56], where response rates were balanced and missing data were due to similar reasons in both groups, to be at a “low” risk of bias. Two studies [53,54] insufficiently reported reasons for losses to follow-up or information on whether attrition was equally distributed between the groups, and we judged the risk of bias to be “unclear”. We judged three studies [50,51,57] to be at a “high” risk of bias due to high attrition, imbalance in numbers, or different reasons for attrition between the two groups.

***Selective reporting***

Three studies [52,53,54] had available study protocols where all prespecified outcomes had been reported and were judged to be at a “low” risk of reporting bias. One study [57] only reported data on QoL domains which were statistically significant, leading to evident reporting bias and precluding inclusion of findings in the meta-analysis. The remaining studies received a judgment of “unclear” risk because no study protocol was available; however, all outcomes mentioned in the aims were reported.

***Other potential sources of bias***

We judged only one study [51] to be at a “high” risk of bias due to the potential risk of contamination. Four studies were rated as having a “low” risk of other potential sources of bias [52,53,54,55], and three studies were rated as having an “unclear” risk due to the timing of outcome questionnaire administration [50] and a possible carry-over effect [56,57].

### 3.4. Effects of Interventions

**Figure 4:**
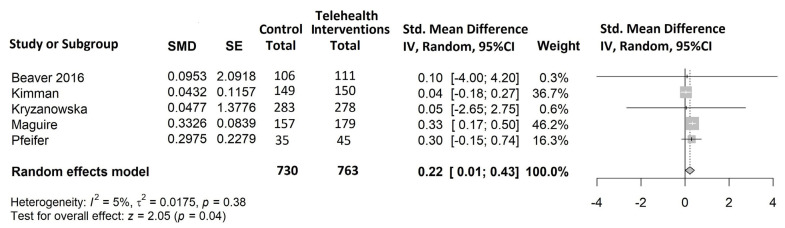
Forest plot of comparison: telehealth intervention versus control; outcome: standardized mean difference for the change from baseline in QoL [52,53,54,55,56].

**Figure 5:**
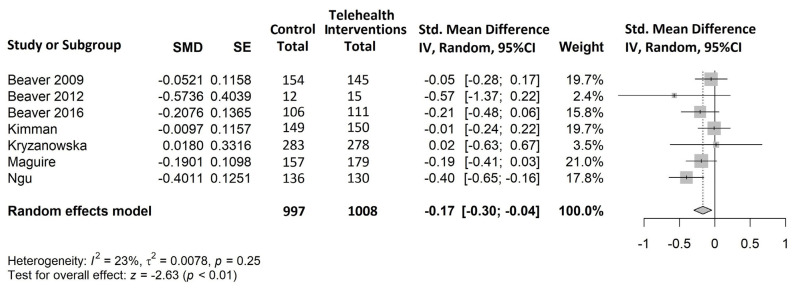
Forest plot of comparison: telehealth intervention versus control; outcome: standardized mean difference for the change from baseline in anxiety [50,51,52,53,54,56,57].

**Figure 6:**
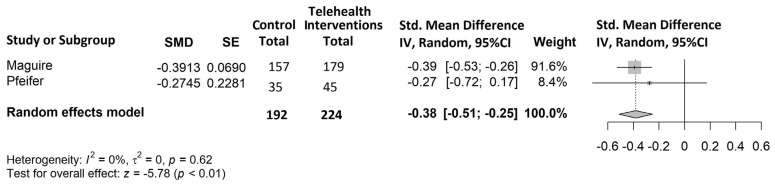
Forest plot of comparison: telehealth intervention versus control; outcome: standardized mean difference for the change from baseline in distress [54,55].

All eight studies selected for the systematic review were included in the meta-analysis, as they all reported at least one of the outcomes of interest. The results of the meta-analysis are shown in Figure 4, Figure 5 and Figure 6.

Quality of Life (Figure 4)

Five studies [52,53,54,55,56] representing 1493/2085 (72%) cancer patients were included in the meta-analysis on this outcome. The study by Ngu et al. [57] also considered QoL but was not included in the meta-analysis because of underreporting. In all included trials, there was an improvement in QoL in the intervention vs. control arm, from mild (SMD = 0.04 in Kimman et al. [52]) to moderate (SMD = 0.33 in Maguire et al. [54]), and the overall estimate was statistically significant (SMD = 0.22, 95% CI 0.01 to 0.43, *p* = 0.04). Furthermore, no heterogeneity was observed.

Anxiety (Figure 5)

Seven studies [50,51,52,53,54,56,57] covering 2005/2085 (96%) patients evaluated this outcome. All studies except Krzyzanowska et al. [53] reported reductions in anxiety, and of these only one recorded a statistically significant effect [57] (SMD = −0.40, 95% CI −0.65 to −0.16). Overall, our meta-analysis identified a small, statistically significant reduction in anxiety levels in favor of telemedicine (SMD = −0.17, 95% CI −0.30 to −0.04, *p* < 0.01). Low heterogeneity was observed (I2 = 23%, *p* = 0.25).

Global distress (Figure 6)

Only two trials [54,55] with 416/2085 (20%) cancer patients evaluated the effects of technology-based interventions on distress and found a reduction in the intervention arm (in Maguire et al. [54]: SMD = −0.39, 95% CI −0.53 to −0.26; in Pfeifer et al. [55]: SMD = −0.27, 95% CI −0.72 to 0.17). Overall, analysis showed a statistically significant moderate effect (SMD = −0.38, 95% CI −0.51 to −0.25, *p* < 0.001) and no heterogeneity.

Depression

The meta-analysis could not be performed as only one study [53] examining this outcome was included.

### 3.5. Subgroup Analyses

All three subgroup analyses planned in the protocol were conducted; however, only two outcomes, QoL and anxiety, could be investigated.

Regarding the effect according to phase of care (patients in active treatment vs. in follow-up), improvement of QoL (Supplementary Figure S1) was statistically significant for patients receiving active treatment, exhibiting medium value of the standard mean difference (three studies, SMD = 0.33, 95% CI 0.17 to 0.48, *p* < 0.001), while no change was observed for patients in follow-up (two studies).

A moderate reduction in anxiety (Supplementary Figure S2) was observed for both phases of care, though it was statistically significant only for patients in follow-up (five studies, SMD −0.18, 95% CI −0.34 to −0.01, *p* = 0.035).

As for the effect according to cancer type, since three out of seven studies focused only on breast cancer [50,52,53] we compared the effect between breast and all other considered cancer types. Both outcomes appear to be improved in patients with other cancer types compared to breast cancer. For QoL (Supplementary Figure S3), a statistically significant improvement was observed for patients affected by cancer types other than breast (three studies, SMD = 0.33, 95% CI 0.17 to 0.48, *p* < 0.001), while no difference was recorded for patients with breast cancer (two studies).

Similarly, anxiety reduction (Supplementary Figure S4) was statistically significant for “other cancer types” (four studies, SMD = −0.27, 90%CI −0.41 to −0.13, *p* < 0.001), while no effect was recorded for patients with breast cancer.

Considering intervention delivery methods, six out of eight studies used the phone [50,51,52,53,56,57] and two studies other modes of administration-website [54] and telehealth messaging device [55]. As for QoL (Supplementary Figure S5), improvement for other modes of administration was statistically significant with a moderate effect (two studies, SMD = 0.33, 95% CI 0.17 to 0.48, *p* < 0.001), while no difference was observed for telephone interventions (three studies).

Regarding anxiety (Supplementary Figure S6), reduction was statistically significant for the telephone (six studies, SMD = −0.17, 90%CI −0.33 to −0.01, *p* = 0.042) vs. other modes of administration (one study), for which the effect was not statistically significant.

## 4. Discussion

### 4.1. Effectiveness of Telehealth Interventions

To the best of our knowledge, this is the first meta-analysis of RCTs investigating the effects of replacing traditional in-person care with forms of telemedicine on psychosocial health of patients with cancer. At the time the work was written, we identified several meta-analyses on this topic [19,20,21,22,23,24,27,28]; however, none was restricted to the evaluation of the impact of virtual care delivered as a replacement of routine, hospital-based in-person care, but they also included telehealth-based psychosocial treatments, which we excluded because they would have altered the measure of the effect of the care modality.

The findings of this meta-analysis indicate that when traditional clinician in-person cancer care is replaced, totally or in part, with technology-based care at a distance, this has no negative influence on patient psychosocial health. On the contrary, improvements were observed for all investigated outcomes. These improvements are clinically relevant, considering that cancer diagnosis and treatment often lead to severe psychosocial consequences. The low heterogeneity we observed furthermore increases confidence in our results.

There may be a number of reasons for these findings. Telehealth interventions may provide cancer patients with faster and easier-to-access knowledge about their disease than traditional healthcare, reducing uncertainty and stress associated with cancer [28]. Additionally, technology may facilitate contact between patients and healthcare professionals, by providing a platform for communication, thus allowing us to respond to patients’ needs more effectively [28] as well as promoting self-management and coping with some cancer-related problems [24]. Moreover, telemedicine can help overcome the obstacle of distance between patients and clinicians, and can reach individuals in remote or rural areas, allowing them to receive necessary health care services [22,24]. Finally, receiving care in a familiar, relaxing environment, away from the hospital setting, may give patients a sense of space to focus on their concerns, thus reducing emotional uneasiness [9].

### 4.2. Subgroup Analysis

Interesting concepts emerged from the three subgroup analyses, although due to the well-established limitations of these investigations (false positives due to multiple comparisons, false negatives due to inadequate power, and limited ability to inform individual treatment decisions [58]), these findings can only be used to build hypotheses, which should be verified in adequate studies. One of these hypotheses is that telemedicine is more beneficial during active treatment with respect to the follow-up phase. A possible explanation for this is that individuals receiving active treatment have a greater need for instructions on how to manage their symptoms, and facilitated contact with clinicians may reduce anxiety and improve quality of life during a particularly challenging phase of the disease trajectory. In fact, QoL trend has been observed to reach its negative peak 3 months after cancer treatment initiation, and then to gradually improve [59]. A second indication emerging from the analysis is that technology-based virtual care does not appear to improve QoL and anxiety for patients with breast cancer, while it does for other cancer types. We could find no explanation for this finding: it is not determined by heterogeneity, and it is not accounted for by a gender effect, since the population included in this meta-analysis is predominantly female (94%). A third hypothesis stems from the observation that telemedicine appears to benefit anxiety and QoL when delivered with modes different from the telephone. Rather than to the type of technology used, this finding may be due to the fact that all studies investigating telephone interventions targeted patients in follow-up, who may not require clinician instructions and contact as much as those in active treatment, as hypothesized above. It is also possible that participants in trials assessing more sophisticated, innovative technologies may differ from those employing the telephone, a device that can be used by everyone without difficulty.

### 4.3. Limitations

This study has some limitations. Firstly, in most studies, psychosocial outcomes were measured as secondary endpoints, which makes our results less robust. Secondly, the quality of included studies is moderate, with only half of them at a low risk of bias and three at a serious risk. Various methodological issues have been detected, mostly relating to attrition and reporting biases. Thirdly, the results of this meta-analysis may not be generalizable to all cancer types and to both sexes, as the included studies mainly concern female neoplasms. Finally, the meta-analysis was based upon summary data, because not all included trials made patient level data available.

## 5. Conclusions

As health care is increasingly being decentralized with the use of communication technologies, one of the main concerns is the possible negative impact that this may have on patients’ psychosocial health. This work responds to this relevant question by showing that telemedicine does not deteriorate but actually ameliorates quality of life, anxiety, or global distress, although many aspects have yet to be clarified. Firstly, although the literature generally suggests good acceptability and satisfaction with telehealth, it is possible that individuals who agree to take part in a trial of telemedicine are already accepting towards this care modality [60]. In this regard, the literature emphasizes a number of implementation issues which must be considered. According to an overview of systematic reviews [61], the most prevalent barrier to telemedicine consists of the lack of evidence to guide telemedicine design, which makes it difficult to adapt interventions for all cancer types, ages, languages, and settings. Furthermore, various obstacles may impede the sustainability of telemedicine, including a lack of available cancer-specific apps, the cost of staff with the necessary skills to deliver it, and the complexity of incorporating patient-centered care into the design [61]. Finally, from a patient perspective, digital illiteracy, as well as inequalities of access to technology, are potential concerns that must be kept in mind whenever the introduction of virtual care systems is considered [60,61]. Thus, it is of paramount importance that the current emphasis on virtual care does not lead to health inequalities.

Future studies, therefore, should aim to identify the types of patients who may benefit the most from telemedicine, and those for whom traditional in-person visits remain the best option, including psychosocial outcomes in their evaluations. Furthermore, it is essential that interventions are appropriately reported to ensure reproducibility, allow researchers to build on research findings, and enable the incorporation of evidence into practice.

## Figures and Tables

**Table 1:** Summary characteristics of included articles.

First Author	Year	Single-Center or Multicenter	Country	No. Patients Randomized and Analyzed in the Meta-Analysis	Age (Years)	Sex (% Female)	Cancer Types	Phase of Care	Intervention vs. Control	Intervention Delivery Method	Outcome of Interest and Instrument: Distress	Outcome of Interest and Instrument: Anxiety	Outcome of Interest and Instrument: Depression	Outcome of Interest and Instrument: Quality of Life
Beaver [50]	2009	Multicenter	United Kingdom	374 randomized (145 intervention arm vs. 154 control arm)	Mean 64 SD 10.6	100%	Breast	Follow-up	Intervention arm: appointments by specialist nurses according to hospital policy, at intervals consistent with hospital follow-up. Control arm: traditional hospital follow-up (consultation, clinical examination, and mammography as per hospital policy). participants were reviewed every three months for two years, six monthly for two years, then annually for a further year.	Telephone	No	Primary: Spielberger state trait anxiety inventory (STAI)	No	No
Beaver [51]	2012	Single-center	United Kingdom	65 randomized (32 intervention arm vs. 33 control arm); 50 analysed (15 intervention arm vs. 12 control arm	Intervention arm: Mean 73.6, SD 7.6; Control arm: Mean 72.4, SD 8.2	42%	Colorectal	Follow-up	Intervention arm: follow-up consultations by a colorectal nurse practitioner using a structured intervention at the same prescribed intervals as the control arm. Control arm: hospital consultations at 6-weeks posttreatment, then 6-monthly intervals for 2 years and annually for a further 3 years	Telephone	No	Primary: Spielberger state trait anxiety inventory (STAI)	No	No
Beaver [56]	2016	Multicenter	United Kingdom	259 randomized (129 intervention arm vs. 130 control arm); 117 analysed (111 intervention arm vs. 106 control arm)	Intervention arm: Median 66, IQR 60–72.5; Control arm: Median 64, IQR 57.8–69	100%	Endometrial	Follow-up	Intervention arm: assessment by gynaecology oncology nurse specialists at intervals consistent with hospital policy at the study locations, mirroring the frequency of scheduled hospital appointments for the control arm. Control arm: hospital based follow-up in accordance with hospital policy at the study locations. This consisted of appointments every 3 or 4 months for the first 2 years post-treatment followed by appointments at decreasing intervals (6-monthly and annually), up to a period of 3–5 years.	Telephone	No	Primary: Spielberger state trait anxiety inventory (STAI)	No	Secondary: European Organization for Research and Treatment (EORTC) QLQ-C30 (version 3) and a specific module for endometrial cancer (QLQ-EN24)
Kimman [52]	2011	Multicenter	The Netherlands	320 randomized (162 intervention arm vs. 158 control arm); 299 analysed (150 intervention arm vs. 149 control arm)	Intervention arm: Mean 55.5, SD 9.0; Control arm: Mean 56.2, SD 10.7	100%	Breast	Follow-up	Intervention arm: interviews by a breast care nurse or nurse practitioner at 3, 6, 9 and 18 months, + mammography and outpatient clinic visit at 12 months. Control arm: hospital follow-up as usual: outpatient clinic visits at the same time points as for the intervention arm, including a clinical visit and a mammography at 12 months.	Telephone	No	Secondary: Spielberger state trait anxiety inventory (STAI)	No	Primary: EORTC Core Quality of Life questionnaire (EORTC QLQ-C30)
Krzyzanowska [53]	2021	Multicenter	Canada	2158 randomized (944 intervention arm vs. 1214 control arm); 580 participant in patient reported outcomes cohort (278 intervention arm vs. 283 control arm)	Median age 55.7	100%	Breast	Active treatment	Intervention arm: nurse-led assessment of common toxicities with two structured follow-up calls during each cycle of chemotherapy using a standardized questionnaire. Control arm: standard of care according to the institution. Typically, standard care involved baseline patient education on chemotherapy and common side effects, and advice to call the cancer centre about symptoms or concerns related to the treatment between visits to the clinic.	Telephone	No	Secondary: generalised anxiety disorder 7 Scale (GAD-7)	Secondary: patient health questionnaire 9 Scale (PHQ-9)	Secondary: EuroQol EQ-5 Dimensions-3 Level (EQ-5D-3L)
Maguire [54]	2021	Multicenter	Austria, Greece, Norway, Republic of Ireland, UK	840 randomised (422 intervention arm vs. 418 control arm); 829 analysed (179 intervention arm vs. 157 control arm)	Mean 52.4, SD 12.2	82%	Breast, Colorectal, Hodgkin’s disease, non-Hodgkin’s lymphoma	Active treatment	Intervention arm: real time, 24 h monitoring and management of chemotherapy toxicity. Patients completed a toxicity self-assessment questionnaire daily and whenever they felt unwell, for up to 6 cycles of chemotherapy. Alerts to clinicians were generated when necessary. Control arm: care as usual at the cancer centre. Participants were advised to contact clinicians through standard mechanisms.	Web site	Secondary: Memorial Symptom Assessment Scale (MSAS) Global Distress Index (GDI)	Secondary: State-Trait Anxiety Inventory—Revised (STAI-R)	No	Secondary: Functional Assessment of Cancer Therapy—General (FACT-G)
Ngu [57]	2020	Single-center	China	385 randomised (191 intervention arm vs. 194 in control arm); 239 anaysed (130 intervention arm vs. 136 control arm)	Intervention arm: Median 50, range 27–84; Control arm: Median 50 range 21–83	100%	Endometrial or ovarian cancer	Follow-up	Intervention arm: interview by research nurses at a 3-monthly interval until 2 years after treatment completion, then 6-monthly for the next 3 years and then annually, + annual clinic follow-up with gynaecologists. Control arm: follow-up according to the local routine schedule, with gynaecological clinic visits for symptom review and clinical examination, performed with the same frequency as the telephone intervention (three months for the first 2 years, then 6-monthly for next 3 years and then annually).	Telephone	No	Secondary: Hospital Anxiety and Depression Scale (HADS)	No	Secondary: European Organization for Research and Treatment (EORTC) QLQ-C30
Pfeifer [55]	2015	Single-center	United States	80 randomized (45 intervention arm vs. 35 control arm)	Intervention arm: Mean 60.73, SD 10.2; Control arm: Mean 59.67, SD 11.8	14%	Head and neck	Active treatment	Intervention arm: the participants were instructed on how to reply to algorithm questions daily, and depending on responses they would receive specific information and recommendations as to when to contact clinicians. Unrelieved symptoms or those targeted as requiring immediate intervention resulted in the coordinator contacting the patient directly by phone and/or contacting clinicians to assure effective and immediate intervention. Control arm: routine care, defined as standard-of-care/assessment-only	Telehealth messaging device connected to telephone line in the patient’s home.	Primary: Memorial Symptom Assessment Scale (MSAS) Global Distress Index (GDI)	No	No	Primary: The Functional Assessment of Cancer Therapy-Head & Neck Scale (FACT-HN)

## Data Availability

The data supporting the findings of this study are available from the corresponding author, RP, upon reasonable request.

## Supplementary Materials

The following supporting information can be downloaded at: https://www.mdpi.com/article/10.3390/cancers15072090/s1, Table S1: Full search terms and search strategies for the three databases; Table S2: Studies excluded after full text review and corresponding reasons; Table S3: Risk of bias domains; Figure S1: Subgroup analysis for effect according to phase of care (patients in active treatment vs. in follow-up) on QoL; Figure S2: Subgroup analysis for effect according to phase of care (patients in active treatment vs. in follow-up) on anxiety; Figure S3: Subgroup analysis for effect according to cancer type (breast vs. other types) on QoL; Figure S4: Subgroup analysis for effect according to cancer type (breast vs. other types) on anxiety; Figure S5: Subgroup analysis for effect according to intervention delivery method (phone vs. other modes) on QoL; Figure S6: Subgroup analysis for effect according to intervention delivery method (phone vs. other modes) on anxiety. Reference [62] has cited in the Supplementary Materials.

## Appendix A. Methods Followed for Risk of Bias Assessment

Risk of bias of included studies was assessed with the Cochrane risk of bias tool for randomized trials (RoB 2) [33] and in accordance with the criteria specified in the Cochrane Handbook for Systematic Reviews of Interventions [34]. This included assessment of the following domains:

- Selection bias (random sequence generation and allocation concealment).
- Performance bias (blinding of participants and personnel).
- Detection bias (blinding of outcome assessment).
- Attrition bias (incomplete outcome data).
- Reporting bias (selective reporting of outcomes).
- Other possible sources of bias (serious issues not captured within the other bias domains such as contamination or inconsistencies with timing of interventions/comparisons).

For selective outcome reporting, we searched for both trial registrations and protocols. Where we were unable to find a trial registration or protocol, we recorded “selective outcome reporting” as unclear. Two review authors (CC, FD) assessed each study as being at “high”, “low”, or “unclear” risk of bias for each item. Review authors were not blinded with respect to study authors, institution, or journal. We used discussion and consensus to resolve any disagreements.

In accordance with the instrument’s indications, the overall risk of bias was judged according to the following criteria: low, if all domains were rated as low risk; some concerns/moderate, if at least one domain was rated as some concerns/moderate but no domain at high risk; high/serious if at least one domain was rated as high/serious, or if the study was judged to have some concerns for multiple domains in a way that substantially lowered confidence in the result.
