# Supplementary material for: Psychosocial Impact of Virtual Cancer Care through Technology: A Systematic Review and Meta-Analysis of Randomized Controlled Trials
Source: Cancers (Basel). 2023 Mar 31;15(7):2090. doi: 10.3390/cancers15072090 (PMC10093026; doi:10.3390/cancers15072090)
Supplement: Supplementary file 1 [file cancers-15-02090-s001.zip › Supplementary table S1_Search strategy.pdf]

MEDLINE (PubMed) (1966 a Maggio 2022)

(((((((("Neoplasms/diagnosis"[Mesh] OR "Neoplasms/physiology"[Mesh] OR "Neoplasms/therapy"[Mesh])) OR ((((neoplasms[Title/Abstract] OR cancer[Title/Abstract] OR neoplasm*[Title/Abstract] OR tumo*[Title/Abstract])))))))))) AND (((((((((("Telemedicine"[Mesh]) OR ("Videoconferencing"[Mesh])) OR ("Remote Consultation"[Mesh]))) OR ((Videoconferencing[Title/Abstract] OR "virtual consultation"[Title/Abstract] OR "videoconsultation"[Title/Abstract] OR telemedicine[Title/Abstract] OR telehealth[Title/Abstract] OR "Virtual planning"[Title/Abstract] OR "Online Patient*"[Title/Abstract] OR teleteaching[Title/Abstract] OR telepathology[Title/Abstract] OR "Virtual Decisional Aid"[Title/Abstract] OR "online patient decision aid"[Title/Abstract] OR Teleconsultation[Title/Abstract] OR Telerevision[Title/Abstract] OR (digital[Title/Abstract] AND pathology[Title/Abstract]) OR "Mobile Health"[Title/Abstract] OR "mHealth"[Title/Abstract] OR "eHealth"[Title/Abstract] OR Videoconferenc*[Title/Abstract] OR "video calls"[Title/Abstract] OR "video-calls"[Title/Abstract] OR telerehabilitation[Title/Abstract] OR "digital training"[Title/Abstract]))))))) OR ((virtual[Title/Abstract] AND (care[Title/Abstract] OR visit*[Title/Abstract] OR assistance[Title/Abstract]))))) AND ((((((((("Meta-Analysis as Topic"[Mesh]) OR "Meta-Analysis"[Publication Type]) OR "meta-analysis[Title] OR systematic review"[Title] OR "meta analy*"[Title] OR "metaanaly*"[Title] OR "Meta-Analysis"[Title])) OR ("systematic literature review"[Title])) OR ("systematic review"[Title/Abstract])) OR (((("Randomized Controlled Trial"[Publication Type]) OR "Randomized Controlled Trials as Topic"[Mesh]) OR ("Clinical Trial" [Publication Type])) OR (randomized[Title/Abstract] OR randomised[Title/Abstract] OR randomly[Title/Abstract]))))))

Embase (Embase.com) (1974 a Maggio 2022)

#1 'neoplasm'/exp/mj
#2 neoplasms OR cancer OR neoplasm* OR tumo*:ti,ab
#3 #1 OR #2
#4 'telemedicine'/exp/mj OR 'videoconferencing'/exp/mj OR 'teleconsultation'/exp/mj
#5 ('information needs' OR 'information provision' OR 'communication skills' OR 'communication skills training' OR videoconferencing OR 'virtual consultation' OR 'videoconsultation' OR 'videoconferenced consultations' OR telemedicine OR telehealth OR 'virtual planning' OR 'digital patient assessment platforms' OR 'digital patient assessment' OR 'online patient*' OR online OR virtual OR platform OR teleteaching OR telepathology OR 'virtual decisional aid' OR 'online patient decision aid' OR teleconsultation OR telerevision OR (digital AND pathology) OR video* OR 'mobile health' OR 'mhealth' OR 'ehealth' OR videoconferenc* OR 'video calls' OR 'video-calls' OR telerehabilitation OR 'digital training' OR 'remote consultation' OR 'tele-consultation') AND 'telephone consultation':ti,ab
#6 (virtual NEXT/2 (care OR visit* OR assistance)):ti,ab
#7 #4 OR #5 OR #6 AND ([cochrane review]/lim OR [systematic review]/lim OR [meta analysis]/lim OR [controlled clinical trial]/lim OR [randomized controlled trial]/lim

PsycInfo (1887 a maggio 2022)

S1 mainsubject(Neoplasms) OR ab(cancer or neoplasms)
S2 mainsubject(Telemedicine) OR mainsubject(Videoconferencing) OR ab(Videoconferencing OR "virtual consultation" OR "videoconsultation" OR telemedicine OR telehealth OR "Virtual planning" OR "Online Patient*" OR teleteaching OR telepathology OR "Virtual Decisional Aid" OR "online patient decision aid" OR Teleconsultation)
