# Supplementary material for: Psychosocial Impact of Virtual Cancer Care through Technology: A Systematic Review and Meta-Analysis of Randomized Controlled Trials
Source: Cancers (Basel). 2023 Mar 31;15(7):2090. doi: 10.3390/cancers15072090 (PMC10093026; doi:10.3390/cancers15072090)
Supplement: Supplementary file 1 [file cancers-15-02090-s001.zip › Supplementary table S2_Studies excluded after full text review.pdf]

Supplementary Table S2: Studies excluded after full text review and corresponding reasons

FIRST AUTHOR	YEAR	REASON FOR EXCLUSION
Absolom [37]	2021	Intervention includes usual care (telemedicine does not replace usual care, either totally or in part)
Bouleftour [38]	2021	Intervention includes usual care (telemedicine does not replace usual care, totally or in part)
Fjell [39]	2020	Intervention includes usual care (telemedicine does not replace usual care, either totally or in part)
Hass [40]	2020	Intervention includes usual care (telemedicine does not replace usual care, totally or in part)
Hoek [41]	2017	Intervention includes usual care (telemedicine does not replace usual care, either totally or in part)
Mooney [49]	2017	Two forms of telemedicine are compared
Traeger [42]	2015	Intervention includes usual care (telemedicine does not replace usual care, either totally or in part)
Viers [48]	2015	Outcomes of interest are not investigated
Walle [43]	2020	Outcomes of interest are not investigated
Wang [47]	2015	Intervention includes usual care (telemedicine does not replace usual care, either totally or in part)
Wheelock [44]	2015	Outcomes of interest are not investigated
Yount [45]	2014	Two forms of telemedicine are compared
Zhang [46]	2013	Outcomes of interest are not investigated
