# Supplementary material for: Psychosocial Impact of Virtual Cancer Care through Technology: A Systematic Review and Meta-Analysis of Randomized Controlled Trials
Source: Cancers (Basel). 2023 Mar 31;15(7):2090. doi: 10.3390/cancers15072090 (PMC10093026; doi:10.3390/cancers15072090)
Supplement: Supplementary file 1 [file cancers-15-02090-s001.zip › Supplementary table S3_Risk of bias domains.pdf]

Supplementary Table S3. Risk of bias summary for RCTs: review authors' judgements about each risk of bias item for each included study.

Author (year)	Random sequence generation (D1)	Allocation concealment (D2)	Blinding of participants and personnel (D3)	Blinding of outcome assessment (D4)	Incomplete outcome data (D5)	Selective reporting (D6)	Other bias (D7)
**Beaver 2009 [50]**	**Low risk**<br><br>Quote: "Allocation sequences were computer generated with randomised permuted blocks."	**Low risk**<br><br>Quote: "Allocation sequences were concealed until interventions were assigned."	**Unclear risk**<br><br>Given the nature of the study, it was not possible to blind participants or personnel.	**Unclear risk**<br><br>Participants were self-assessors and were not blinded but the likely direction of bias cannot be predicted.	**High risk**<br><br>A *Fig 1 Flow of participants* shows more participants in the telephone follow-up did not receive the intervention or wanted to change group and were lost to follow-up. Furthermore, participants with no data were dropped with the assumption that data were missing at random, although it was reported that ITT analysis was performed.	**Unclear risk**<br><br>No protocol available, however all outcomes mentioned in the aims were reported	**Unclear risk**<br><br>Quote: "For practical reasons we could not administer outcome questionnaires before randomisation. We sent initial questionnaires to patients immediately after randomisation, a minimum of three months before their next appointment."<br><br>Comment: baseline measures can be influenced due to lack of blinding but the direction of bias is difficult to predict.
**Beaver 2012 [51]**	**Low risk**<br><br>Quote: "Consenting individuals were randomized to either hospital or telephone follow-up by a computerized system."	**Low risk**<br><br>Quote: "Allocation sequences were concealed until interventions were assigned."	**Unclear risk**<br><br>Given the nature of the study, it was not possible to blind participants or personnel.	**Unclear risk**<br><br>Participants were self-assessors and were not blinded but the likely direction<br><br>of bias cannot be predicted	**High risk**<br><br>Quote: "Not all participants provided complete data on the primary outcome measures. Only 12 (48%) hospital and 15 (60%) telephone participants provided complete data on the STAI at baseline and follow-up."<br><br>Comment: 25% of the participants in both groups were lost to follow-up. Although similar numbers were lost to follow-up the reasons were different.	**Unclear risk**<br><br>No protocol available, however all outcomes mentioned in the aims were reported.	**High risk**<br><br>Quote: "The same nurse conducted some of the hospital appointments and all the telephone appointments. Although the nurse only used the structured telephone intervention with patients randomized to the telephone arm, contamination is possible and would need to be avoided in a main trial."

**Beaver 2016** [56]	**Low risk**<br><br>Quote: "Patients were randomly assigned (1:1) to HFU or TFU using a computer-based system."	**Low risk**<br><br>Quote: "Patients were randomly assigned (1:1) to HFU or TFU using a computer-based system."	**Unclear risk**<br><br>Given the nature of the study, it was not possible to blind participants or personnel.	**Unclear risk**<br><br>Participants were self-assessors and were not blinded but the likely direction of bias cannot be predicted.	**Low risk**<br><br>Similar numbers and reasons for loss to follow-up	**Unclear risk**<br><br>No protocol available, however all outcomes mentioned in the aims were reported.	**Unclear risk**<br><br>Quote: "It was not possible to recruit all participants immediately after their first post-treatment outpatient appointment. Although 51% of women were <1 year post surgery, many would have experienced a number of hospital outpatient appointments, and this may have biased the outcomes. Given that women would have experienced at least one hospital appointment prior to recruitment it is not possible to state when the introduction of TFU would be most beneficial or if the findings are generalizable to the first follow-up appointment."<br><br>Comment: possible carry-over effect may attenuate the effect of the intervention.

**Kimman 2011** [52]	**Low risk**<br><br>Quote: "Randomization by minimization was performed by the independent Comprehensive Cancer Center Limburg using a computerized randomization program (ALEA)."	**Low risk**<br><br>Quote: "Randomization by minimization was performed by the independent Comprehensive Cancer Center Limburg using a computerized randomization program (ALEA)."	**Unclear risk**<br><br>Given the nature of the study, it was not possible to blind participants or personnel.	**Unclear risk**<br><br>Participants were self-assessors and were not blinded but the likely direction of bias cannot be predicted.	**Low risk**<br><br>Similar numbers and reasons for loss to follow-up and similar response rates in both groups	**Low risk**<br><br>Protocol available and all outcomes described in the protocol have been reported	**Low risk**<br><br>We detected no other bias.
**Krzyzanowska 2021** [53]	**Low risk**<br><br>Quote: “The cluster randomisation was performed at the Ontario Clinical Oncology Group, Hamilton, Ontario”	**Low risk**<br><br>Quote: “The cluster randomisation was performed at the Ontario Clinical Oncology Group, Hamilton, Ontario”	**Unclear risk**<br><br>Given the nature of the study, it was not possible to blind participants or personnel.	**Unclear risk**<br><br>Participants were self-assessors and were not blinded but the likely direction of bias cannot be predicted.	**Unclear risk**<br><br>Quote: “The patient reported outcome questionnaires were completed by 580 patients, a subset of the total study population (27%), who might not have been representative of the full cohort”.<br>It also reported "Twenty two participants in the patient reported outcomes study did not complete a questionnaire (five in the intervention arm and 14 in the control arm)”.<br>Although the number of loss to follow-up is small (4%), it is not correctly reported, it is biased against the control arm and the reasons for the lost to follow-up are unclear.	**Low risk**<br><br>Protocol available and all outcomes described in the protocol have been reported	**Low risk**<br><br>We detected no other bias.
**Maguire 2021** [54]	**Low risk**<br><br>Quote: “Surrey Clinical Trials Unit randomised patients remotely and independently using the Promasys system”	**Low risk**<br><br>Quote: “Research staff accessed the Promasys system remotely and completed a web based electronic case report form for the participant, and the allocation was assigned automatically”	**Low risk**<br><br>Quote: ”The nature of the intervention meant that blinding of patients was not possible, but patients were blinded to study hypotheses.”	**Unclear risk**<br><br>Quote: ”Blinding of evaluators was achieved, as participants’ allocation was concealed from the statistical analysis team.”	**Unclear risk**<br><br>For patient-reported outcomes (secondary endpoints), the number of responders is not indicated	**Low risk**<br><br>Protocol available and all outcomes described in the protocol have been reported	**Low risk**<br><br>We detected no other bias.
**Ngu 2020** [57]	**Low risk**<br><br>Quote ”Block randomisation with a randomised size of 4 was used. Generation of	**Low risk**<br><br>Quote ”Block randomisation with a randomised size of 4 was used. Generation of	**Unclear risk**<br><br>Given the nature of the study, it was not possible to blind participants or	**Unclear risk**<br><br>Participants were self-assessors and were not blinded but the likely direction of bias cannot be	**High risk**<br><br>Quote: " With regard to QoL outcomes, for the 0–2 years subset, questionnaires from a total	**High risk**<br><br>Quote: “Only selected variables from the questionnaires and symptom checklist with	**Unclear risk**<br><br>Quote: ” were we limited by a small sample size and a patient population with a large skew towards

	randomisation schedule was performed by a person independent of the recruitment, and the randomisation schedule was kept securely by the randomiser.”	randomisation schedule was performed by a person independent of the recruitment, and the randomisation schedule was kept securely by the randomiser.”	personnel.	predicted.	of 96 women (47 in nurse and 49 in doctor group) were available for analysis.”<br><br>Comment: 75% of the participants in both groups were lost to follow-up. Although similar numbers were lost in the two arms, the reasons for attrition/exclusions are not reported.	significant difference between the groups in any of the 3 subsets were presented (Table 2).”<br><br>Comment: evident underreporting as only favorable results for the intevention are presented	early-stage gynaecological malignancies, but also by the fact that the median time after treatment at study entry was 6 years. As a result, women enrolled in the study would have had multiple experiences of hospital-based oncologist-led appointments, potentially biasing the outcomes of this study in favour of the nurse-led model and, given that 80% of gynaecological cancer recurrences occur within the first two years following treatment”<br><br>Comment: possible carry-over effect may attenuate the effect of the intervention.
**Pfeifer 2015** [55]	**Unclear risk**<br><br>Quote: “After securing informed consent, the study coordinator consulted a randomization grid which considered treatment modalities to ascertain if the participant would be assigned to treatment or control.”<br>It also reported “Our patients differed demographically from national norms in head and neck cancer by gender (86% men in our study vs 73% men nationally)”	**Unclear risk**<br><br>Quote: “After securing informed consent, the study coordinator consulted a randomization grid which considered treatment modalities to ascertain if the participant would be assigned to treatment or control.”	**Unclear risk**<br><br>Given the nature of the study, it was not possible to blind participants or personnel.	**Unclear risk**<br><br>Participants were self-assessors and were not blinded but the likely direction of bias cannot be predicted.	**Low risk**<br><br>Similar numbers and reasons for loss to follow-up and similar response rates in both groups	**Unclear risk**<br><br>No protocol available, however all outcomes mentioned in the aims were reported.	**Low risk**<br><br>We detected no other bias.

RoB was assessed using the Cochrane “risk of bias” tool [*Higgins JPT* et al,*The Cochrane Collaboration’s tool for assessing risk of bias in randomised trials BMJ 2011; 343 doi: https://doi.org/10.1136/bmj.d5928 (Published 18 October 2011) Cite this as: BMJ 2011;343:d5928*]
