# Supplementary figures and images for: Psychosocial Impact of Virtual Cancer Care through Technology: A Systematic Review and Meta-Analysis of Randomized Controlled Trials
Source: Cancers (Basel). 2023 Mar 31;15(7):2090. doi: 10.3390/cancers15072090 (PMC10093026; doi:10.3390/cancers15072090)

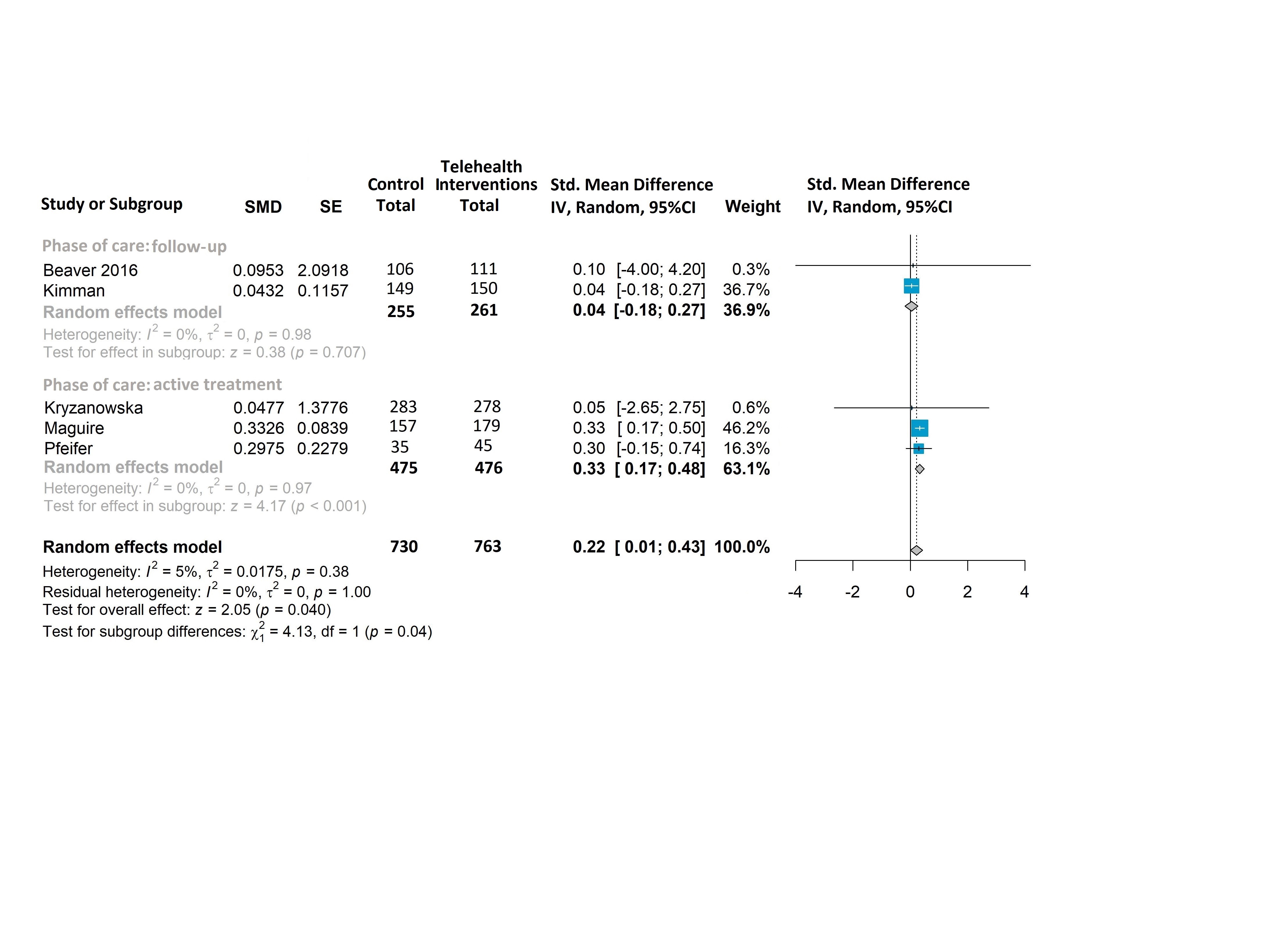

Supplement: Supplementary file 1 [file cancers-15-02090-s001.zip › FigureS1_subgroup_QoL_phase.jpg]

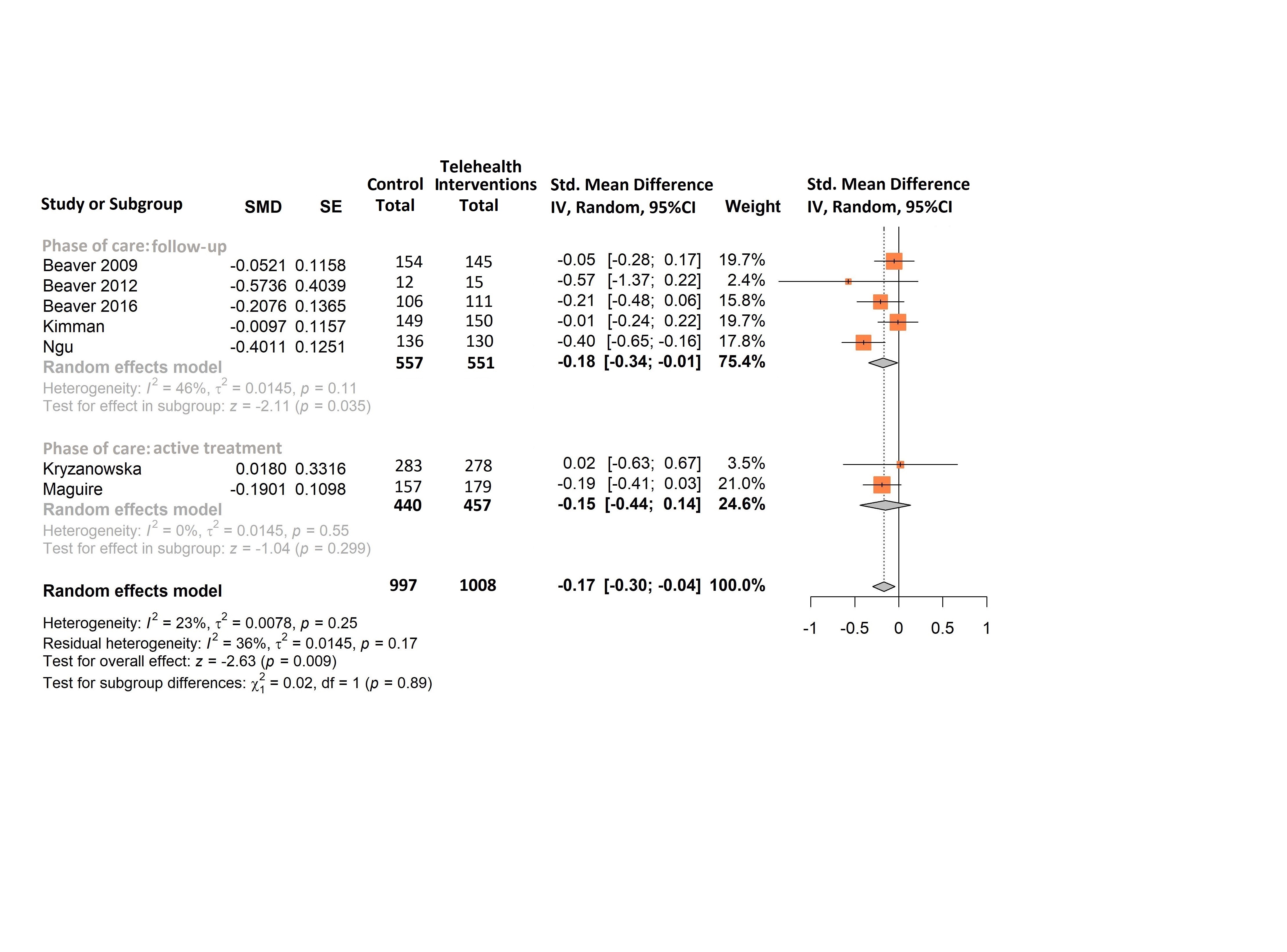

Supplement: Supplementary file 1 [file cancers-15-02090-s001.zip › FigureS2_subgroup_anxiety_phase.jpg]

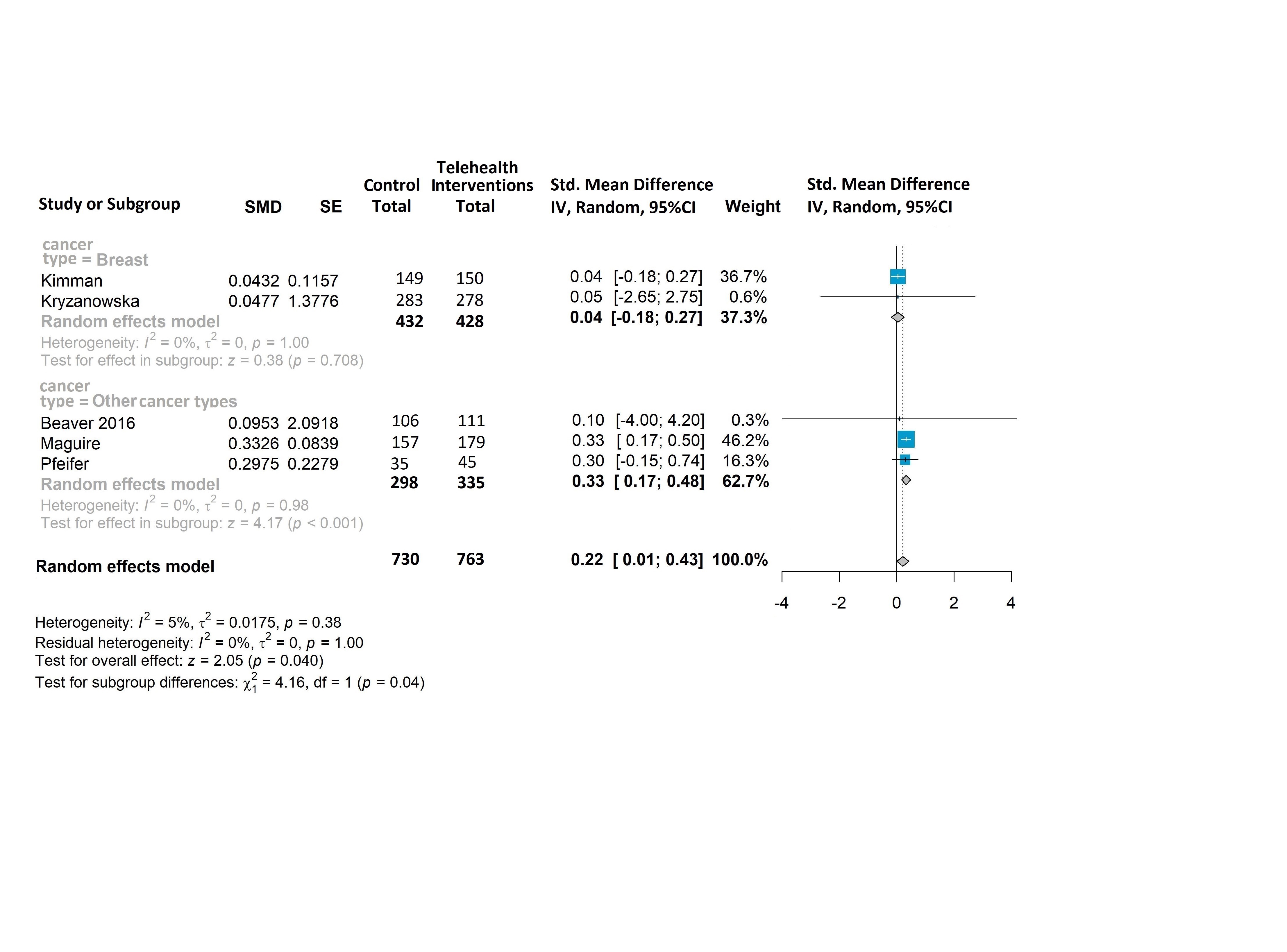

Supplement: Supplementary file 1 [file cancers-15-02090-s001.zip › FigureS3_subgroup_QoL_cancer.jpg]

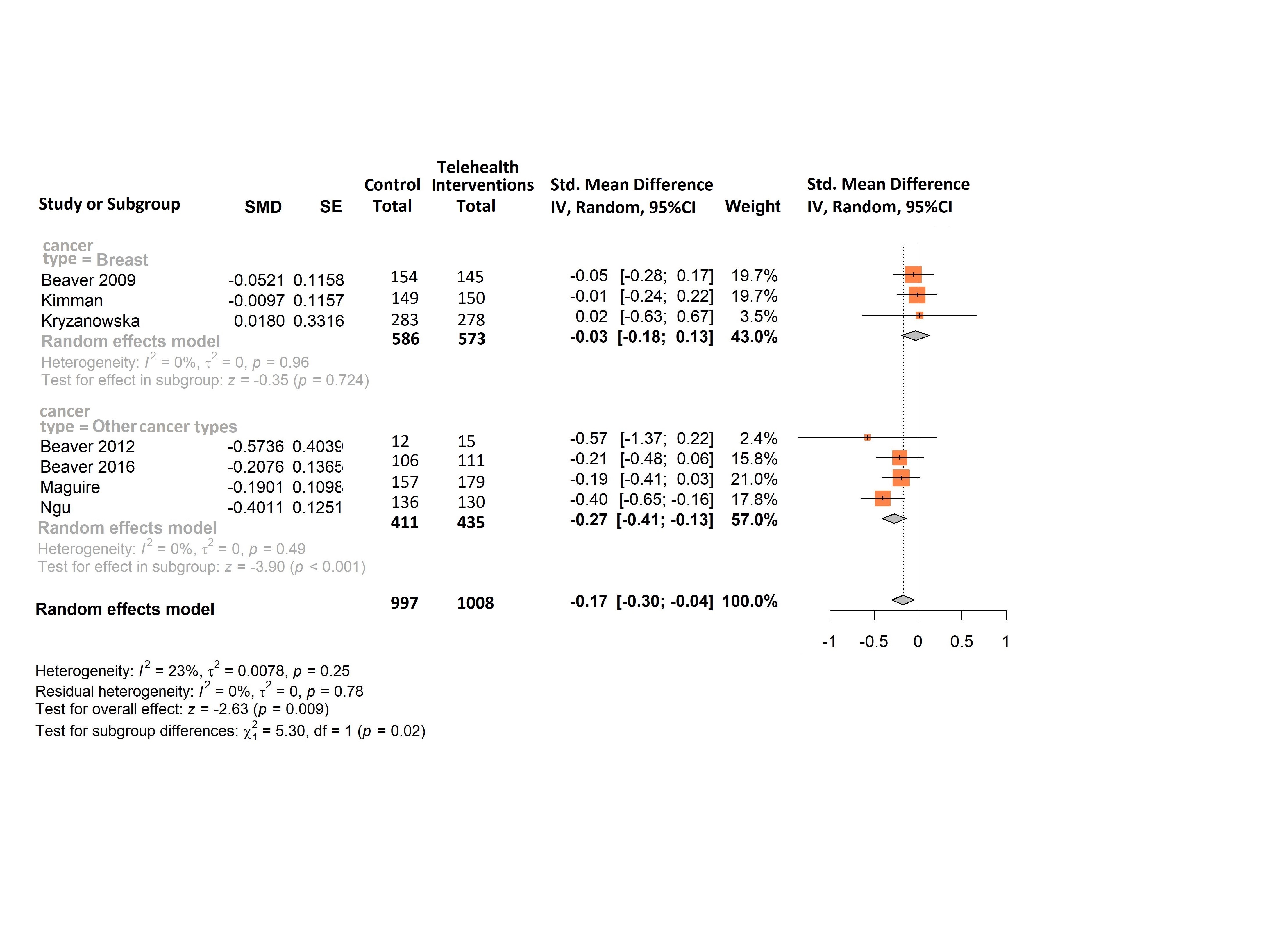

Supplement: Supplementary file 1 [file cancers-15-02090-s001.zip › FigureS4_subgroup_anxiety_cancer.jpg]

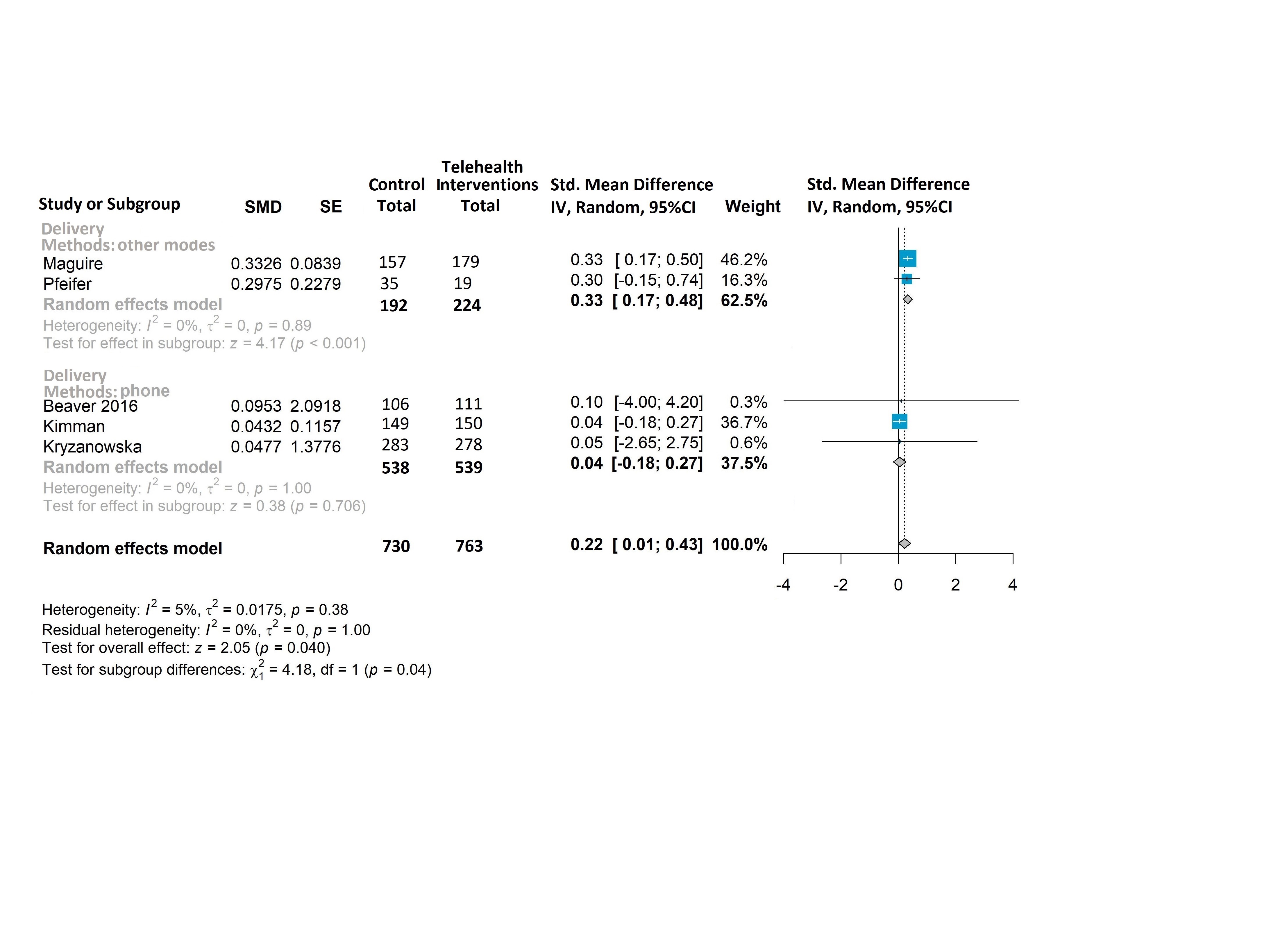

Supplement: Supplementary file 1 [file cancers-15-02090-s001.zip › FigureS5_subgroup_QoL_delivery.jpg]

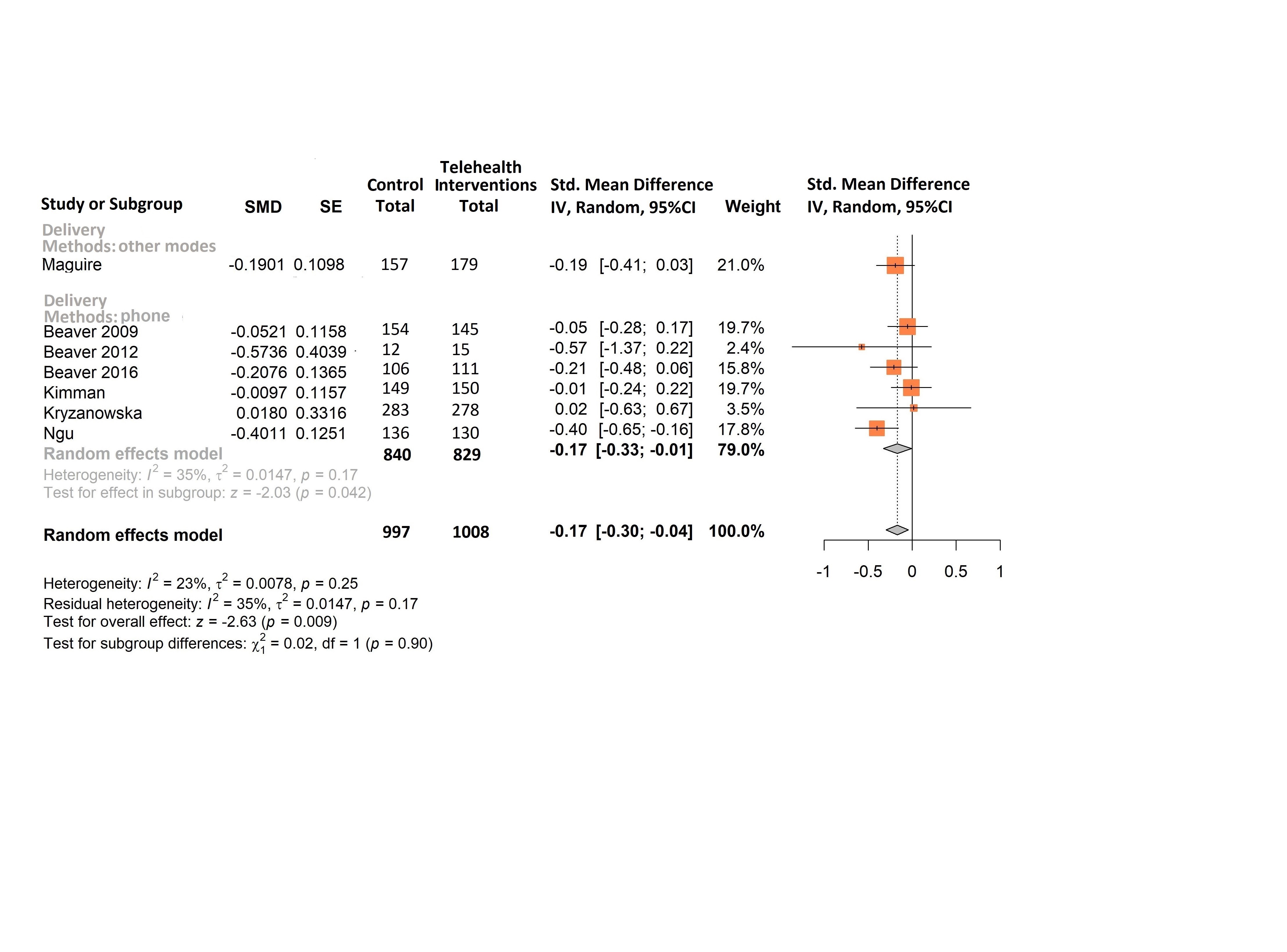

Supplement: Supplementary file 1 [file cancers-15-02090-s001.zip › FigureS6_subgroup_anxiety_delivery.jpg]
